# Development and Cross‐Cultural Validation of the Vietnamese Version of the Disaster Prevention Consciousness Scale: A Cross‐Sectional Study

**DOI:** 10.1002/hsr2.71669

**Published:** 2025-12-27

**Authors:** Naomi Akiyama, HaAnh Nguyen, Kan Shimazaki, PHAM Hong Ha, Shihoko Kajiwara

**Affiliations:** ^1^ Graduate School of Nursing, Medical School Department of Health Sciences Nagoya City University Nagoya Japan; ^2^ Graduate School of Economics Nagoya City University Nagoya Japan; ^3^ Department of Human Factors Engineering and Environmental Design Faculty of Biology‐Oriented Science and Technology Kinokawa Japan; ^4^ Hue International General Hospital Hue Vietnam; ^5^ Center for postgraduate clinical training and career development Nagoya University Hospital Nagoya Japan

**Keywords:** cross‐cultural validation, disaster prevention consciousness scale, Japan, Vietnam

## Abstract

**Background and Aims:**

The Japanese Disaster Prevention Consciousness Scale (J‐DPCS) addresses all natural disasters, making it more adaptable to foreign populations. However, certain items in the scale refer to disaster preparedness measures which may not be emphasized in countries where infrastructure development for disaster prevention is less prioritized, hence the need for modification. We aimed to validate the 20‐item J‐DPCS and develop and evaluate the Vietnamese Disaster Prevention Consciousness Scale (V‐DPCS) through a cross‐sectional survey in Japan and Vietnam.

**Methods:**

We surveyed 618 Japanese adults and 496 Vietnamese adults. The J‐DPCS was validated using factor analysis, internal consistency was assessed with Cronbach's αand McDonald's ω, and concurrent validity was examined using the Spearman's rank‐order correlations and bootstrapping. For the V‐DPCS, item difficulty and discrimination were estimated using the Item Response Theory (Two‐Parameter Logistic model). Item‐total correlations with bootstrapping were used to assess reliability. Differences in response scales between Japan and Vietnam were adjusted, and a confirmatory factor analysis was conducted.

**Results:**

Factor analysis revealed a five‐factor structure for both versions, with acceptable internal consistency for most factors. Factor 1 (0.85), 2 (0.79), and 3 (0.80) maintained strong reliability compared to their original values (0.906, 0.863, and 0.868, respectively). Factor 4 had a low reliability coefficient of 0.60 and 0.55 in the original and 20‐item version, respectively. Factor 5 consisted of four items with similar Cronbach's alpha coefficients.

**Conclusion:**

The 20‐item J‐DPCS demonstrated strong validity and reliability. The validated core model of the V‐DPCS, consisting of 12 items across Factors 1–3, is suitable for cross cultural application. However, Factors 4 and 5 require further development to enhance measurement stability. These findings underscore the complexities of adapting disaster prevention scales across cultures and support the utility of the core V‐DPCS structure in diverse settings.

## Summary

- What's known
  ◦ The Japanese Disaster Prevention Consciousness Scale (J‐DPCS) is adaptable to foreign populations. However, certain items referring to disaster preparedness measures may not be emphasized in countries where infrastructure development for disaster prevention is less prioritized, hence, the need for modification.
- What's new
  ◦ We developed a Vietnamese Disaster Prevention Consciousness Scale (V‐DPCS). Compared to the original 40‐item version, the shortened 20‐item version demonstrated a higher cumulative contribution rate.
- Clinical implications
  ◦ The validated core model of the V‐DPCS, consisting of 12 items across Factors 1–3, is suitable for cross cultural application.

## 1 Introduction

Disasters such as earthquakes, storms, floods, and droughts claim ~40,000–50,000 lives annually [1]. Japan is highly disaster‐prone, accounting for 20.8% of global earthquakes (magnitude ≥ 6), 7.0% of active volcanoes, 0.4% of disaster‐related deaths, and 18.3% of global damages [2]. Disaster awareness is essential for citizen protection and requires: (1) reinforcing cultural values and daily routines, (2) integrating preparedness into everyday life, and (3) enhancing self‐efficacy by showing how daily skills can be applied in disaster situations [3]. The Japanese government emphasizes that disasters can strike anyone, anywhere, at any time, and promotes integrating preparedness into daily life [4]. Globally, the *Sendai Framework for Disaster Risk Reduction 2015–2030* provides a basis for prevention‐oriented, people‐centered, and culturally inclusive policies [5], highlighting four priorities—understanding risk, strengthening governance, investing in resilience, and enhancing preparedness—emphasizing shared responsibility among governments, communities, and individuals.

The number of international migrant workers has risen steadily. In 2019, 272 million migrants were reported worldwide, including 169 million workers [6]. In Japan, the number of foreign workers reached 2,048,675 in 2024, up from 1,822,725 in 2023; Vietnamese workers formed the largest group (518,364; 25.3%), followed by Chinese (19.4%) and Filipino workers (11.1%) [7]. Disaster impacts vary by physical, psychological, social, economic, and environmental vulnerabilities [8], which are pronounced among older adults, pregnant women, children, patients, low‐income groups, caregivers, persons with disabilities, and immigrants [9, 10]. Strengthening networks and sharing risk among diverse stakeholders before disasters is essential for effective disaster risk management in multicultural contexts [11]. The Great Hanshin‐Awaji Earthquake (1995) caused 174 deaths among foreign nationals, and 41 died in the Great East Japan Earthquake (2011). As the number of foreign residents and visitors to Japan grows—3,781,200 international visitors in January 2025, a 40.6% increase from 2019 [12]—disaster preparedness for non‐Japanese residents becomes increasingly vital. Although 69% of foreign residents participated in disaster drills, most were conducted in Japanese [13].

A disaster awareness scale developed by the National Research Institute for Earth Science and Disaster Resilience (NIED) is used to assess awareness after drills [14]. This 20‐item scale, derived from an earlier 40‐item version [15], is widely used, yet its development and validity remain unclear. Most studies on preparedness in Japan focus on earthquakes [16]. The DPCS covers multiple natural disasters, making it more adaptable to foreign populations. However, some items, e.g., earthquake‐resistant buildings or seawalls—may be less relevant in regions where infrastructure for disaster prevention is limited, necessitating modification.

This study aimed to validate the 20‐item Japanese Disaster Prevention Consciousness Scale (J‐DPCS), translate it into Vietnamese, and develop the Vietnamese version (V‐DPCS).

## 2 Methods

### 2.1 Study Design

Permission was obtained from the developers of the 40‐item DPCS, to validate the 20‐item version of the V‐DPCS [15]. (Shimazaki ＆ Ozeki, 2022).

A cross‐sectional survey was conducted to develop the V‐DPCS.

## 3 Data

### 3.1 Data for Developing the 20‐Item J‐DPCS

We surveyed 618 adults (mean age: 46.2 years) from Japan, chosen randomly from an online survey panel of ~2.3 million individuals registered with Macromill Inc. There were no geographical or attribute‐specific limitations. The 20 items published as the Japanese version are shown in Table 1.

**Table 1:** The Disaster‐prevention consciousness scale.

**Factor 1: Imagination of the damages**		
	*Q1*	*I have a concrete image of how people behave at the time of occurrence of a disaster*.
	*Q3*	*I have a concrete image of the necessary materials at the time of occurrence of a disaster*.
	*Q5*	*I have a concrete image of how the town would go at the time of occurrence of a disaster*.
	*Q19*	*I have a concrete image of how I should respond at the time of occurrence of a disaster*.
**Factor 2: Sense of crisis to current measures against disaster**		
	*Q6*	*If disaster should occur once, I think it would be in serious trouble*.
	*Q12*	*It is not strange that disasters would occur tomorrow*.
	*Q13*	*I think it would be difficult to mitigate the damage of disaster only through the efforts of individuals*.
	*Q17*	*I think disaster prevention should not be completed within one's own region, but in coordination with other regions*.
**Factor 3: Other directedness**		
	*Q4*	*I want to have various friends*.
	*Q16*	*I like to communicate with others*.
	*Q18*	*I like the place where people gather*.
	*Q20*	*I want to do something for others*.
**Factor 4: Indifference to disaster prevention**		
	*Q2*	*I do not want to do anything that does not benefit me*.
	*Q9*	*I only think about what would likely happen around me*.
	*Q11*	*Usually, I do not think about a disaster*.
	*Q15*	*I think physical measures such as earthquake strengthening and improvement of seawalls would be enough to manage earthquakes*.
**Factor 5: Anxiety**		
	*Q7*	*I think I am a worrywart*.
	*Q8*	*I often feel anxious*.
	*Q10*	*Once I think about a disaster, I imagine various patterns of damage*.
	*Q14*	*I always care about the danger around me*.

*Note* [13].

Factor 1: Ability to understand disaster impacts through concrete imagination.

Factor 2: Awareness of risk and inadequacy of current disaster countermeasures.

Factor 3: Willingness to support others and participate in mutual assistance during disasters.

Factor 4: Low perceived relevance or motivation for disaster preparedness.

Factor 5: Emotional sensitivity to disaster risk that may drive or hinder preparedness.

### 3.2 The Procedure for Translating the 20‐Item Japanese Version of DPCS Into Vietnamese

The quality of a scale depends on the selection of qualified translators [17]. Two independent bilingual translators with master's degrees, fluent in Japanese and Vietnamese and familiar with Japanese culture after over 5 years of residence, conducted the translation. Translator A translated the original Japanese version into Vietnamese, and Translator B independently back‐translated it into Japanese. Conversely, Translator B translated the scale into Vietnamese, and Translator A back‐translated it into Japanese. This cross–back translation ensured linguistic validity without direct discussion between translators. Discrepancies, including linguistic nuances and cultural differences, were reviewed with the original authors. Experts in disaster medicine and preparedness evaluated the translation's accuracy. For items related to infrastructure less familiar to Vietnamese respondents, minor adaptations improved clarity while retaining the original meaning.

The 6‐point response format was converted to a 4‐point scale for easier smartphone use. Linear interpolation (1 → 1.00, 2 → 1.60, …, 6 → 4.00) preserved proportional spacing. Prior psychometric studies show that reducing response options (e.g., 4 vs. 6 points) does not compromise reliability or validity [18] and maintains factor structures within the 4–7 range [19]. These findings supported adopting a 4‐point format to maintain psychometric comparability and enhance usability.

As the translators had higher educational backgrounds than the intended respondents, literacy differences were considered. One co‐author obtained feedback from Vietnamese speakers in Japan, and minor revisions were made. Based on their comments, the terms “worry” and “anxiety” were refined for clarity.

### 3.3 Data for Evaluating the Reliability and Validation of the Vietnamese Version of the DPCS

Vietnamese who participated in the Vietnam Festa completed the questionnaire. In December 2024, we recruited participants from hospitals and universities in Hanoi, Vietnam. Sampling was not based on age or region of origin.

## 4 Analysis

### 4.1 To Develop the 20‐Item J‐DPCS

We used factor analysis to identify the factor structure and measure disaster awareness [15]. Principal axis factoring with promax (oblique) rotation was employed, and items with factor loadings of ≥ 0.4 were retained. Cronbach's α and McDonald's ω were calculated to assess the internal consistency of each factor, and the cumulative contribution ratio was computed to evaluate the total variance explained by the extracted factors. We validated the results by comparing them with scales from previous studies [20, 21, 22, 23].

### 4.2 To Develop the V‐DPCS

#### 4.2.1 Descriptive Statistics Summarized Participants’ Demographics

Item Response Theory (IRT) using a two‐parameter logistic model estimated item difficulty and discrimination. Nonparametric item–total correlations (Spearman's ρ) were calculated for each subscale, and 95% confidence intervals were obtained via bootstrapping with 2500 resamples. Internal consistency was assessed using Cronbach's α and McDonald's ω.

To compare Vietnamese disaster scale scores between Japanese and Vietnamese participants, response‐scale differences were adjusted: Japanese 6‐point responses were converted to a 4‐point scale (1 = 1.00, 2 = 1.60, 3 = 2.20, 4 = 2.80, 5 = 3.40, 6 = 4.00).

Confirmatory factor analysis (CFA) was performed in R (lavaan package) and JMP version 18.0, with significance set at *p* < 0.05. Analyses were run in RStudio 2025.09.1 + 401 “Cucumberleaf Sunflower” Release (20de356561bd58a6d88927cce948bd076d06e4ca, 2025‐09‐23) for windows　Mozilla/5.0 (Windows NT 10.0; Win64; x64) AppleWebKit/537.36 (KHTML, like Gecko) RStudio/2025.09.1 + 401 Chrome/138.0.7204.185 Electron/37.2.6 Safari/537.36, Quarto 1.7.32. Convergent and discriminant validity were evaluated according to the criteria proposed by Fornell and Larcker [24], using factor loadings, composite reliability (CR), and average variance extracted (AVE) to assess the adequacy of the measurement model.

### 4.3 Ethical Considerations

This study was approved by the Institutional Ethics Committee (Approval No. R6‐2‐004). An online anonymous survey was conducted in Vietnamese, with detailed study information provided.

### 4.4 Participation Was Voluntary, and Informed Consent Was Obtained Through an Opt‐in Checkbox

## 5 Results

### 5.1 Developing the J‐DPCS

Five distinct factors were extracted: imagination of the damage (Factor 1), sense of crisis to current measures against disaster (Factor 2), other directedness (Factor 3), indifference to disaster prevention (Factor 4), and anxiety (Factor 5). The specific factors, loadings, and cumulative contribution ratios differ slightly (Table 2). The 20‐item version explained 50.9% of the total variance, with Cronbach's α ranging from 0.55 to 0.85 across factors, indicating acceptable reliability. AVE for a representative construct was 0.57, supporting convergent validity. In addition, the single‐factor CFA model showed McDonald's ω (total) values of 0.62 for the Japanese six‐point version and 0.65 for the four‐point version, indicating moderate overall internal consistency. Correlations with previous studies ranged from −0.24 to 0.45 (Table 3), showing patterns consistent with earlier findings. Overall, the 20‐item model demonstrated satisfactory reliability, validity, and cross‐cultural applicability.

**Table 2:** The 20 item J‐DPCS consisting of four items per five subscales (*n* = 618).

Item	Factor 1	Factor 2	Factor 3	Factor 5	Factor 4	Cronbach's α (McDonal's ω)	Cumulative contribution ratio
Q1	0.780	0.026	0.170	0.085	0.000	0.85 (0.85)	14.22
Q3	0.748	0.077	0.129	0.093	0.010		
Q19	0.733	0.026	0.171	0.002	−0.032		
Q5	0.687	−0.004	0.191	0.131	−0.006		
Q6	−0.016	0.777	0.035	0.229	−0.025	0.79 (0.79)	25.47
Q12	0.075	0.694	0.018	0.199	0.002		
Q17	0.110	0.661	0.182	0.211	−0.043		
Q13	0.012	0.551	−0.013	0.101	−0.189		
Q16	0.151	0.094	0.801	0.015	0.034	0.80 (0.82)	36.20
Q18	0.197	−0.051	0.739	0.010	−0.055		
Q4	0.114	0.011	0.733	0.043	0.049		
Q20	0.279	0.212	0.468	0.094	0.147		
Q8	0.097	0.247	0.013	0.781	−0.048	0.76 (0.78)	45.16
Q7	−0.058	0.303	0.007	0.685	−0.022		
Q10	0.298	0.196	0.119	0.500	−0.047		
Q14	0.433	0.150	0.047	0.459	0.011		
Q2	−0.005	−0.060	0.163	−0.097	0.592	0.55 (0.52)	50.89
Q9	−0.091	−0.083	−0.028	0.085	0.544		
Q11	0.233	−0.111	−0.004	0.212	0.495		
Q15	−0.379	0.353	−0.104	−0.016	0.425		

*Note:* To examine the structural equivalence of the 40‐item Japanese version of the DPCS, an exploratory factor analysis (EFA) using promax rotation was conducted. J‐Abbreviation: DPCS, Japanese Disaster Prevention Consciousness Scale.

**Table 3:** The correlations between the five factors with previous studies.

Previous studies	Factor 1	Factor 2	Factor 3	Factor 4	Factor 5
	Coefficient [95% CI] *p* value	Coefficient [95% CI] *p* value	Coefficient [95% CI] *p* value	Coefficient [95% CI] *p* value	Coefficient [95% CI] *p* value
Arai et al.(*n* = 539)	0.377 [0.299, 0.455] < 0.001	0.123 [0.032, 0.206] 0.004	0.214 [0.125, 0.299] < 0.001	0.077 [−0.011, 0.163] 0.076	−0.151 [−0.231, −0.064] < 0.001
Ishihara and Matsumura (*n* = 539)	0.442 [0.369, 0.511] < 0.001	−0.020 [−0.106, 0.064] 0.651	0.335 [0.252, 0.413] < 0.001	0.045 [−0.043, 0.127,] 0.302	−0.203 [−0.291, −0.117] < 0.001
Hada et al. (Factor 1) (*n* = 539)	0.175 [0.081, 0.264] < 0.001	0.287 [0.199, 0.368] < 0.001	0.283 [0.196, 0.363] < 0.001	0.119 [0.037, 0.201] 0.006	−0.240 [−0.329, −0.151] < 0.001
Hada et al. (Factor 2) (*n* = 539)	0.043 [−0.046, 0.129] 0.322	0.285 [0.200, 0.362] < 0.001	0.076 [−0.009, 0.160] 0.080	0.237 [0.154, 0.314] < 0.001	−0.136 [−0.221, −0.048] 0.002
Motoyoshi. (Factor 1) (*n* = 617)	0.404 [0.330, 0.472] < 0.001	0.043 [−0.034, 0.122] 0.291	0.249 [0.170, 0.329] < 0.001	−0.113 [−0.192, −0.030] 0.005	0.043 [−0.041, 0.130] 0.281
Motoyoshi. (Factor 2) (*n* = 617)	0.350 [0.275, 0.422] < 0.001	0.039 [−0.044, 0.117] 0.330	0.451 [0.382, 0.519] < 0.001	−0.005 [−0.093, 0.077] 0.898	−0.044 [−0.127, 0.041] 0.277

*Note:* The correlations between the previous studies and the five factors were analyzed using Spearman's rank‐order correlation coefficients (ρ). The 95% confidence intervals (CI) were obtained via the bootstrap method with 2500 resamples. Due to missing data, entries without responses were excluded from the analysis.

### 5.2 Flow Diagram of the V‐DPCS Development

**Figure 1:**
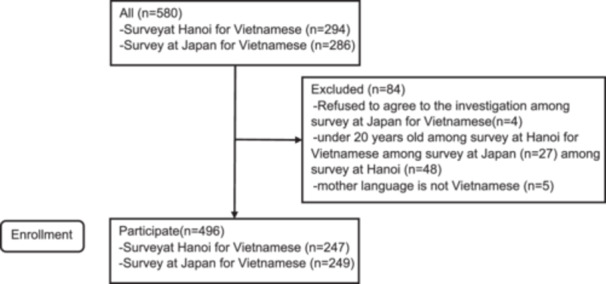
Flow diagram of the Japanese Disaster Prevention Consciousness Scale (J‐DPCS) and its adaptation process into the Vietnamese Disaster Prevention Consciousness Scale (V‐DPCS).

We recruited 580 Vietnamese individuals surveyed in Hanoi (*n* = 294) and Japan (*n* = 286); 84 were excluded (Figure 1). Finally, 496 participants were included in the analysis.

### 5.3 Participant Characteristics

The participants’ demographic characteristics are summarized below (Table 4). Of 496 participants, 249 (50.2%) and 247 (49.8%) were surveyed in Japan and Hanoi, respectively. Among them, 470 participants (94.8%) reported having experienced a natural disaster. Overall, 260 participants (52.4%) were men and 227 (45.8%) were female. Most participants (468 participants, 94.4%) were aged 20–39 years, 224 (45.2%) had no history of living in Japan, 149 (30.0%) had lived in Japan for 1–5 years, 33 (6.7%) had stayed for 6–10 years, 11 (2.2%) had lived in Japan for over 10 years, 177 (35.7%) were students, and 183 (36.9%) were company employees. Percentages may not total 100% because of rounding.

**Table 4:** Characteristics of participants in the V‐DPCS study (*n* = 496).

Variable	*n* (%)
Experience of natural disaster	470 (94.8)
Storm	412 (83.1)
Flood	137 (27.6)
Earthquake	126 (25.4)
Heavy Rain	37 (7.5)
Heavy Snow	26 (5.2)
Landslide	23 (4.6)
Tsunami	9 (1.8)
Volcanic Eruption	1 (0.2)
Sex	
Men	260 (52.4)
Female	227 (45.8)
None	9 (1.8)
Age, years	
20–39 years	468 (94.4)
40–59 years	27 (5.4)
60 years and above	1 (0.2)
Length of stay in Japan	
No residence history in Japan	224 (45.2)
Less than 6 months	16 (3.2)
Within 1 year	63 (12.7)
1–5 years	149 (30.0)
6–10 years	33 (6.7)
Over 10 years	11 (2.2)
Occupation	
Student	177 (35.7)
Housewife	27 (5.4)
Government employee	6 (1.2)
Company employee	183 (36.9)
Self‐employed	28 (5.6)
Technical intern trainee	72 (14.5)
Others	3 (0.6)
Survey place	
Japan	249 (50.2)
Hanoi	247 (49.8)

*Note:* Percentages may not total 100% because of rounding.

Abbreviation: V‐DPCS, Vietnamese Disaster Prevention Consciousness Scale

### 5.4 Factor Analysis of the V‐DPCS

Table 5 shows the IRT results and reliability analysis for the 20‐item V‐DPCS. Factors 1–3 had strong discrimination values (> 1) and moderate‐to‐low difficulty, indicating good differentiation and accessibility, whereas Factors 4 and 5 showed weaker discrimination and lower internal consistency. Item–total correlations ranged from 0.33 to 0.52 (*p* < 0.001), supporting adequate relationships between each item and the total score. Cronbach's α coefficients for the five subscales ranged from 0.59 to 0.78, suggesting acceptable internal consistency. Although slightly lower reliability was observed for Factors 1, 2, and 4 in the Vietnamese version compared with the Japanese version, the overall factor structure remained consistent, confirming the cross‐cultural applicability of the 20‐item V‐DPCS.

**Table 5:** The 20‐item V‐DPCS consisting of four items per five subscales (*n* = 496).

Item	Item Response Test (IRT)		Item‐Total (IT) correlation			Cronbach's α (McDonald's ω)
	Difficulty	Discrimination	Coefficient	95%CI	*p* value	
**Factor 1**						
Q1	1.164	1.051	0.464	[0.382, 0.539]	< 0.001	0.72(0.73)
Q3	0.847	1.111	0.462	[0.377, 0.532]	< 0.001	
Q5	0.511	1.390	0.447	[0.360, 0.522]	< 0.001	
Q19	0.729	1.132	0.421	[5e‐33, 4.9e‐15]	< 0.001	
**Factor 2**						
Q6	1.182	1.253	0.391	[2.4e‐29, 1.9e‐12]	< 0.001	0.70(0.71)
Q12	0.982	1.579	0.454	[0.377, 0.528]	< 0.001	
Q13	1.089	1.266	0.363	[0.274, 0.449]	< 0.001	
Q17	1.132	1.862	0.436	[0.356, 0.515]	< 0.001	
**Factor 3**						
Q4	1.055	1.462	0.502	[0.424, 0.570]	< 0.001	0.78 (0.77)
Q16	1.320	1.416	0.520	[0.445, 0.586]	< 0.001	
Q18	1.222	0.994	0.506	[0.434, 0.579]	< 0.001	
Q20	1.756	2.005	0.513	[0.432, 0.582]	< 0.001	
**Factor 4**						
Q2	0.361	0.422	0.327	[0.240, 0.406]	< 0.001	0.59 (0.56)
Q9	0.900	0.824	0.355	[0.261, 0.433]	< 0.001	
Q11	1.184	0.631	0.332	[0.242, 0.412]	< 0.001	
Q15	0.232	0.392	0.352	[0.263, 0.427]	< 0.001	
**Factor 5**						
Q7	0.515	1.159	0.439	[0.360, 0.511]	< 0.001	0.65 (0.62)
Q8	0.344	0.899	0.380	[0.298, 0.457]	< 0.001	
Q10	0.834	1.727	0.328	[0.242, 0.415]	< 0.001	
Q14	0.758	1.743	0.340	[0.245, 0.427]	< 0.001	

*Note:* NoIRT was analyzed using the two‐parameter logistic (2PL) model.

Assuming a nonparametric distribution, the item‐total correlations were calculated using Spearman's rank correlation coefficient. The 95% confidence intervals (CI) were obtained via the bootstrap method with 2500 resamples.

Abbreviation: The V‐DPCS, Vietnamese Disaster Prevention Consciousness Scale.

### 5.5 Comparison between the J‐DPCS and the V‐DPCS Scores

Differences in factor structures were observed between the survey results for Japanese and Vietnamese participants. General tendencies in response patterns were noted. The factors with the largest differences in response tendencies were Factor 1 (Difference score = −2.6, *p* <0.001), Factor 3 (Difference score = −3.6, *p* < 0.001), and Factor 5 (Difference score = −1.6, *p *< 0.001). No significant differences between Factor 2 and Factor 4 existed. An examination of the median differences for each item revealed that the medians for Japanese and Vietnamese participants differed by 0.2 and 0.8 points (Table 6, Figure 3_SuppInfo).

**Table 6:** Comparison between J‐DPCS and V‐DPCS scores.

Item	Japanese (n = 618)		Vietnamese (*n* = 496)		Difference	*p* value
	Median	IQRs	Median	IQRs		
**Factor 1**						
Q1	2.2	0.6	3.0	1.0	−0.8	< 0.001
Q3	2.2	0.6	3.0	1.0	−0.8	< 0.001
Q5	2.2	0.6	3.0	1.0	−0.8	< 0.001
Q19	2.2	0.6	3.0	2.0	−0.8	< 0.001
**Factor 2**						
Q6	3.4	0.6	3.0	1.75	0.4	0.376
Q12	2.8	0.6	3.0	2.0	−0.2	< 0.001
Q13	3.4	1.2	3.0	2.0	0.4	0.806
Q17	2.8	0.6	3.0	1.0	−0.2	< 0.001
**Factor 3**						
Q4	2.2	0.6	3.0	2.0	−0.8	< 0.001
Q16	2.2	1.2	3.0	1.0	−0.8	< 0.001
Q18	2.2	0.6	3.0	2.0	−0.8	< 0.001
Q20	2.8	0.6	3.0	1.0	−0.8	< 0.001
**Factor 4**						
Q2	2.8	0.6	2.0	1.0	0.8	0.369
Q9	2.2	1.2	2.0	1.0	0.2	0.037
Q11	2.2	0.6	2.0	1.0	0.2	0.004
Q15	2.2	0.6	2.0	1.0	0.2	0.020
**Factor 5**						
Q7	2.2	0.6	3.0	1.0	−0.8	< 0.001
Q8	2.8	0.6	3.0	1.0	−0.2	0.031
Q10	2.2	0.6	3.0	2.0	−0.8	< 0.001
Q14	2.8	0.6	3.0	2.0	−0.2	< 0.001
Factor 1	9.4	2.4	12.0	3.0	−2.6	< 0.001
Factor 2	12.4	2.4	12.0	3.0	0.4	0.281
Factor 3	9.4	2.4	13.0	3.0	−3.6	< 0.001
Factor 4	9.4	2.4	9.0	3.0	0.4	0.943
Factor 5	9.4	1.8	11.0	3.0	−1.6	< 0.001

*Note:* The Vietnamese version uses a 1–4‐point scale, so the Japanese version's 1–6‐point scale was converted to a 4‐point scale and presented in a table.

IQRs means interquartile ranges (IQRs). Difference represented the median difference between the Vietnamese and Japanese versions of the DPCS.

The Wilcoxon rank‐sum test was used to compare the DPCS scores between Japanese and Vietnamese participants. Since Q2, Q9, Q11, and Q15 under Factor 4 were reverse‐coded items, their scores were reversed by subtracting each from 5. The Factor 4 total score was then obtained by subtracting the sum of these four items from 20. This method of scoring followed the procedure adopted in J‐DPCS.

Abbreviations: J‐DPCS, Japanese Disaster Prevention Consciousness Scale; V‐DPCS, Vietnamese Disaster Prevention Consciousness Scale.

### 5.6 Improving the Measurement Model of the V‐DPCS

**Figure 2:**
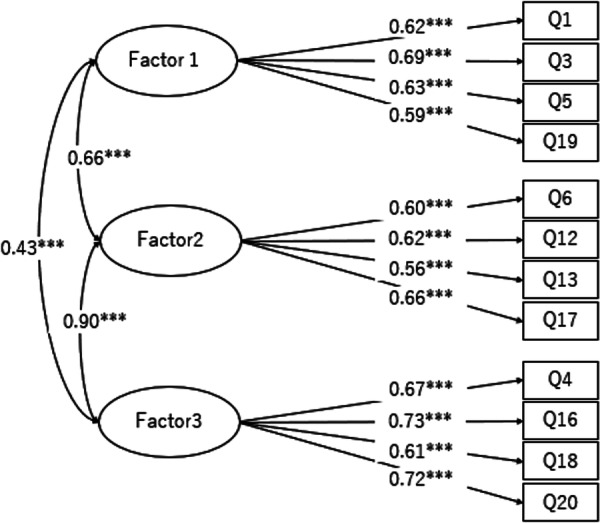
Revised measurement model of the Vietnamese Disaster Prevention Consciousness Scale (Revised V‐DPCS; n = 496) (χ^2^ = 106.410, d.f.=51.000, *p* < 0.001, CFI = 0.967, IFI = 0.967, RMSEA = 0.047) V‐DPCS, Vietnamese Disaster Prevention Consciousness Scale.

The 20‐item V‐DPCS was initially developed to replicate the five‐factor structure of the Japanese version. However, while the 20‐item model demonstrated acceptable but suboptimal fit indices (χ²=913.40, *p* < 0.001; CFI = 0.823; TLI = 0.797; RMSEA = 0.086), the CFA based on the Vietnamese sample indicated limited construct validity and insufficient reliability in Factors 4 and 5. This revised 12‐item model consisting of Factors 1–3 was subsequently tested and demonstrated an acceptable model fit (χ^2^ = 106.410, d.f.=51.000, *p* <0.001, CFI = 0.967, IFI = 0.967, RMSEA = 0.047) (Figure 2) and in the Japanese sample (χ² = 151.857, d.f. = 51, *p* < 0.001, CFI = 0.963, IFI = 0.963, RMSEA = 0.057) (Figure 4_SupplInfo). All factor loadings were above 0.4, indicating adequate relationships between the observed variables and their respective factors. Cronbach's α coefficients for the three retained subscales ranged from 0.70 to 0.78, and McDonald's ω coefficients were 0.73, 0.71, and 0.77 for Factors 1–3, respectively, with an overall ω total of 0.83 for the 12‐item model. These results indicate acceptable to good internal consistency. Although the AVE values were slightly below 0.50 (Factor 1 = 0.40, Factor 2 = 0.37, Factor 3 = 0.46), the factor loadings (0.47–0.98) indicated sufficient convergent validity. Overall, the refined model achieved improved construct validity and a more parsimonious structure suitable for cross‐cultural application.

## 6 Discussion

### 6.1 Validity of the 20‐item J‐DPCS

Compared to the original 40‐item version, the shortened 20‐item version demonstrated a higher cumulative contribution rate (47.4% vs. 50.9%). Of five subfactors, the internal consistency of Factor 4 was lower in the 20‐item version (0.547 vs. 0.603), while that of the other factors remained similar. For Factor 4, the internal consistency remained relatively low (0.60) in the original scale, suggesting inadequate representation of the intended construct.

Attempts were made to develop disaster awareness scales in Japan; all of them have relatively low cumulative contribution rates of 30–50% [20, 21]. Contrarily, resilience scales, demonstrated high reliability and accuracy [16, 25]. Some resilience scales include questions about specific behaviors, which reduce variability in responses. Contrarily, DPCS excludes behavioral aspects, lowering the discriminative power of the questions. The discriminative power of the questions included in Factor 4 of the V‐DPCS was below 1, indicating that responses were less distinct.

### 6.2 Validity of the 20‐item V‐DPCS

Compared with the original Japanese version, the subscale reliability coefficients were uniformly lower, indicating reduced internal consistency. Cronbach's α for Factors 4 (0.59) and 5 (0.65) were low, even relative to the Japanese version (0.55 and 0.77). Consequently, Factors 4 and 5 were provisionally excluded to form a revised 12‐item model. Future research should reconstruct these factors to enhance cultural relevance and psychometric robustness.

For Factor 4, item discrimination indices below 1.0 indicated limited ability to distinguish respondents with differing levels of this trait. Although item–total correlations were acceptable, the construct may not reflect realities in Vietnam, where disasters are part of daily life. Most participants were aged 20–39 years, unlike the older Japanese sample (mean 42.6 years). Because awareness of disaster prevention varies by age and experience [26], the younger demographic, combined with Vietnam's high hazard exposure [27, 28], may have influenced perceptions of “indifference.” Future revisions should assess tendencies to downplay preparedness or to prioritize other daily concerns over disaster readiness. For Factor 5, items may have lacked specificity. Although frequent exposure can reduce immediate anxiety, major events such as typhoons or floods can cause lasting trauma [29]. More concrete items—e.g., “I worry about whether my family can evacuate during a disaster” or “I feel anxious when imagining my house destroyed”—may capture latent anxieties more effectively. Differentiating between acute disaster‐related anxiety and general everyday worries is essential to refine this factor. These issues are linked to cultural concepts. Hofstede [30] posits that societies manage uncertainty through culturally reinforced institutions such as family, school, and state. Vietnamese people exhibit greater tolerance for uncertainty than Japanese people (The Culture Factor, 2023). Thus, Vietnamese respondents may display a coping style that accepts disasters as they occur rather than articulating related emotions, consistent with literature on cultural and social‐cognitive influences on preparedness (Paton, 2003). Cross‐cultural studies also show that cultural heritage and everyday hazard exposure shape preparedness and risk perception [29, 31]. While Factors 4 and 5 require refinement, Factors 1–3 were well measured. Disaster prevention awareness is inherently abstract and harder to operationalize than behavioral or resilience constructs. Nonetheless, the scale remains useful for research and practice owing to its conceptual breadth and applicability, particularly for evaluating disaster drills in formal and informal care settings.

### 6.3 Differences in Disaster Prevention Awareness and Implications for Preparedness Strategies

Compared to Japanese participants, Vietnamese participants scored higher on Factors 1, 3, and 5 DPCS. Since Factors 4 and 5 may not have been appropriately measured due to cultural differences, they were excluded from the analysis.

Even though Hanoi experiences fewer disasters compared to other regions in Vietnam, it is likely that participants possess a higher level of disaster‐related imagination than those in Japan. During a disaster, it is difficult to overcome challenges without the cooperation of the entire community. The Japanese government emphasizes the importance of mutual assistance (kyōjo) as a key principle in its disaster management policies (Cabinet Office Japan, 2023). Vietnamese culture permits a high degree of individual autonomy within a collectivist framework, partly due to its tradition of power distance. This is different from individualism and can be interpreted as a reflection of the Vietnamese temperament, which characterized by a high tolerance for uncertainty, allows greater acceptance of others’ autonomous behaviors. We did not investigate the perception of Vietnamese residents in Japan towards others and society. Although Japan is promoting the acceptance of Vietnamese technical intern trainees, collaboration between the trainees and local Japanese communities remains limited. As Wialdi et al. [32] noted, disaster preparedness is culturally rooted. Further, Fathani et al. [11] emphasized the importance of pre‐disaster collaboration among diverse stakeholders [32]; (Fathani, 2023). Building on these insights, transferring Japan's disaster‐related cultural practices to foreign residents, and strengthening cooperation with local communities are essential for future preparedness. In line with previous studies, considering disaster preparedness measures in Japan that leverage the cultural values of Vietnamese residents is necessary [31].

### 6.4 Limitations

This study has several limitations. First, most participants were young adults (20–39 years), many of whom were students, which may have introduced selection bias in the Vietnamese cohort. This age bias limits generalizability because disaster‐prevention consciousness may differ among older adults or adolescents. However, this reflects the demographic profile of Vietnamese in Japan: 69.1% of visitors are under 30 years [33], and residents in their 20s–30 s account for 491,479 individuals, representing 95.2% of all Vietnamese nationals [34]. In Japan, university students have relatively low disaster preparedness [14]. Future studies should include a broader age range. Second, urban–rural differences were not analyzed. Socioeconomic and health conditions vary regionally in Vietnam [35], and future research should consider these differences and population mobility. Third, some items in Factors 4 and 5 were too abstract, and several participants reported difficulty responding. Finally, discriminant validity was not examined for the Japanese or Vietnamese versions. A criterion for high preparedness is needed, as self‐initiated preparedness increases after disasters [36]. Higher DPCS scores after the Kumamoto Earthquake suggest that the scale captures shift in preparedness awareness (Ozeki et al., 2018). Future studies should address discriminant validity.

## 7 Conclusion

This study underscores the importance of adapting and validating disaster prevention consciousness scales for use in different cultural settings. Future research should focus on refining the scale to enhance its sensitivity and reliability, ensuring its applicability across diverse populations and disaster types. Future studies should consider controlling potential confounding variables, such as previous disaster experience, education level, and access to disaster preparedness training, as socio economic factors and personal experiences can significantly influence disaster preparedness awareness. Building organizational resilience through capacity development, including innovation, human resource management, and knowledge management, may offer useful insights for disaster preparedness in multicultural settings [37]. Special attention should be given to reconstructing Factors 4 and 5 to better reflect the cultural context of Vietnamese respondents, and to contribute to the development of a more comprehensive, cross‐culturally valid measurement tool for disaster prevention consciousness.

## Author Contributions

**Naomi Akiyama:** conceptualization, data curation, formal analysis, funding acquisition, investigation, methodology, project administration, resources, validation, visualization, writing – original draft, writing – review and editing **HaAnh Nguyen:** conceptualization, data curation, formal analysis, investigation **Kan Shimazaki:** conceptualization, supervision, investigation, methodology **PHAM Hong Ha:** conceptualization, supervision **Shihoko Kajiwara:** data curation, formal analysis, methodology, validation, visualization, writing – original draft, writing – review and editing. All authors have read and approved the final version of the manuscript.

## Conflicts of Interest

The authors declare no conflicts of interest.

## Transparency Statement

The lead author Naomi Akiyama affirms that this manuscript is an honest, accurate, and transparent account of the study being reported; that no important aspects of the study have been omitted; and that any discrepancies from the study as planned (and, if relevant, registered) have been explained.

## Supporting information


**Supplementary Figure 3:** Comparison of Disaster Prevention Consciousness Scale (DPCS) scores between Japanese and Vietnamese participants, including total and subscale scores Comparison between J‐DPCS and V‐DPCS scores J‐DPCS, Japanese Disaster Prevention Consciousness Scale; V‐DPCS, Vietnamese Disaster Prevention Consciousness Scale. **Supplementary Figure 4:** Measurement model of the Japanese Disaster Prevention Consciousness Scale (J‐DPCS; n = 618).

## Data Availability

The data that support the findings of this study are available from the corresponding author upon reasonable request. Naomi Akiyama had full access to all the data in this study and takes complete responsibility for the integrity of the data and the accuracy of the data analysis.
