# Supplementary material for: Development and Cross‐Cultural Validation of the Vietnamese Version of the Disaster Prevention Consciousness Scale: A Cross‐Sectional Study
Source: Health Sci Rep. 2025 Dec 27;9(1):e71669. doi: 10.1002/hsr2.71669 (PMC12743725; doi:10.1002/hsr2.71669)
Supplement: Supplementary file 1 — Supplementary Figure 3: Comparison of Disaster Prevention Consciousness Scale (DPCS) scores between Japanese and Vietnamese participants, including total and subscale scores Comparison between J‐DPCS and V‐DPCS scores J‐DPCS, Japanese Disaster Prevention Consciousness Scale; V‐DPCS, Vietnamese Disaster Prevention Consciousness Scale. Supplementary Figure 4: Measurement model of the Japanese Disaster Prevention Consciousness Scale (J‐DPCS; n = 618). [file HSR2-9-e71669-s001.docx]

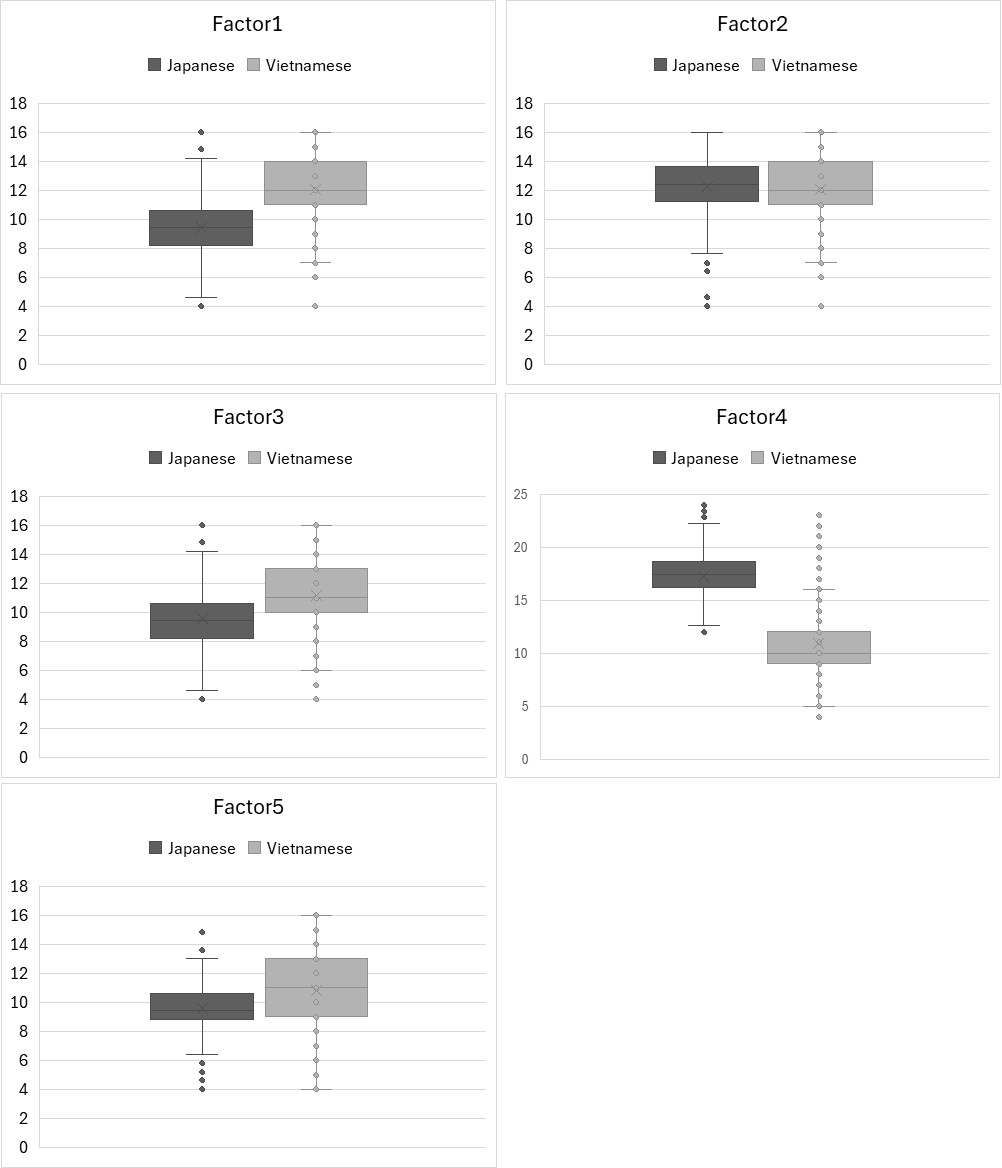


Supplementary Figure 3. Comparison of Disaster Prevention Consciousness Scale (DPCS) scores between Japanese and Vietnamese participants, including total and subscale scores

Comparison between J-DPCS and V-DPCS scores J-DPCS, Japanese Disaster Prevention Consciousness Scale; V-DPCS, Vietnamese Disaster Prevention Consciousness Scale


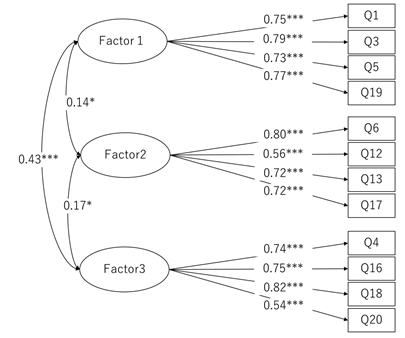


Supplementary Figure 4. Measurement model of the Japanese Disaster Prevention Consciousness Scale (J-DPCS; n = 618)
(χ² = 151.857, d.f. = 51.000, p < 0.001, CFI = 0.963, IFI = 0.963, RMSEA = 0.057)

Multi-group confirmatory factor analyses were conducted to examine measurement invariance across the Japanese and Vietnamese samples.
The change in CFI (ΔCFI) between the two groups was 0.004, which is below the recommended cutoff of 0.01 for establishing measurement invariance (Cheung & Rensvold, 2002).

**Reference**

G. W. Cheung and R. B. Rensvold, “Evaluating Goodness‐of‐Fit Indexes for Testing Measurement Invariance,” *Structural Equation Modeling: A Multidisciplinary Journal* 9, no. 2 (2002): 233–255, https://doi.org/10.1207/S15328007SEM0902_5.
